# PhyloSophos: a high-throughput scientific name mapping algorithm augmented with explicit consideration of taxonomic science, and its application on natural product (NP) occurrence database processing

**DOI:** 10.1186/s12859-023-05588-3

**Published:** 2023-12-14

**Authors:** Min Hyung Cho, Kwang-Hwi Cho, Kyoung Tai No

**Affiliations:** 1 Bioinformatics and Molecular Design Research Center (BMDRC), 209, Veritas A Hall, Yonsei University, 85 Songdogwahak-ro, Yeonsu-gu, Incheon, 21983 Republic of Korea; 2 School of Systems Biomedical Science, Soongsil University, Seoul, 06978 South Korea; 3 Department of Integrative Biotechnology and Translational Medicine, 214, Veritas A Hall, Yonsei University, 85 Songdogwahak-ro, Yeonsu-gu, Incheon, 21983 Republic of Korea

**Keywords:** Scientific name, Taxonomic reference, Identifier standardization, Natural product, Natural product occurrence database, Database integration

## Abstract

**Background:**

The standardization of biological data using unique identifiers is vital for seamless data integration, comprehensive interpretation, and reproducibility of research findings, contributing to advancements in bioinformatics and systems biology. Despite being widely accepted as a universal identifier, scientific names for biological species have inherent limitations, including lack of stability, uniqueness, and convertibility, hindering their effective use as identifiers in databases, particularly in natural product (NP) occurrence databases, posing a substantial obstacle to utilizing this valuable data for large-scale research applications.

**Result:**

To address these challenges and facilitate high-throughput analysis of biological data involving scientific names, we developed PhyloSophos, a Python package that considers the properties of scientific names and taxonomic systems to accurately map name inputs to entries within a chosen reference database. We illustrate the importance of assessing multiple taxonomic databases and considering taxonomic syntax-based pre-processing using NP occurrence databases as an example, with the ultimate goal of integrating heterogeneous information into a single, unified dataset.

**Conclusions:**

We anticipate PhyloSophos to significantly aid in the systematic processing of poorly digitized and curated biological data, such as biodiversity information and ethnopharmacological resources, enabling full-scale bioinformatics analysis using these valuable data resources.

**Supplementary Information:**

The online version contains supplementary material available at 10.1186/s12859-023-05588-3.

## Introduction

The appropriate utilization of unique identifiers is of growing importance in the rapidly advancing fields of bioinformatics and systems biology [1]. With an ever-increasing volume of biological data from diverse sources, accurate identification and tracking of biological entities, such as genes, proteins, and pathways, are essential for seamless data integration, comprehensive analysis, and reproducibility of research findings [2]. Unique identifiers, like accession numbers, play a pivotal role in providing standardized and unambiguous representation of biological entities across multiple databases and platforms, enabling efficient data exchange and collaborative research efforts [3]. A coherent and standardized system is crucial for recognizing underlying biological patterns and extracting meaningful insights from vast amounts of high-throughput data [4]. Thus, researchers can reveal hidden correlations and relationships, propelling our understanding of complex biological processes and leading to groundbreaking discoveries.

Although scientific names serve as universally accepted identifiers for biological species, they exhibit inherent limitations that render them insufficient in various aspects [5, 6]. These limitations stem from the absence of certain desirable qualities expected from an ideal database identifier, including stability, uniqueness, persistence, and convertibility [1, 7, 8]. Scientific names, often undergo revisions and updates as new taxonomic information emerges: this dynamic nature poses challenges in maintaining consistency across different resources and databases. Furthermore, the sheer diversity of organisms and the existence of synonyms, homonyms, and common names associated with species further complicate the verification and standardization of scientific names [9]. The lack of universal adoption of taxonomic standards also contributes to the complexity of cross-referencing and integrating information from multiple sources [10].

Scientific names are particularly pertinent when it comes to information concerning natural products (NPs) and metabolites [11]. Throughout history, NPs have been recognized for their inherent therapeutic properties and have consistently contributed to advancements in disease treatments [12]. Remarkably, more than half of the recently approved medications can trace their origins back to NPs or their derivatives, underscoring their profound significance in modern medicine [13]. NPs encompass a diverse range of chemical compounds known for their unique biological activities and chemical properties. Intriguingly, these NPs appear to have undergone an evolutionary process that optimizes their properties, prioritizing superior availability and bioactivity. This remarkable attribute makes them particularly promising as potential sources of oral drugs that surpass the limitations imposed by Lipinski's rule of five, opening up new avenues for drug development and providing improved therapeutic options for patients [14].

The diligent efforts of natural product researchers have yielded a wealth of valuable information on the diverse range of compounds found in specific species. However, integrating and organizing this data poses significant challenges, including the standardization of heterogeneous data and the need for accurate annotation [15]. Furthermore, the lack of real-time assessment and updates for the majority of NP occurrence information often results in names being superseded by new 'canonical' names [16], leaving disagreements between databases. This issue is exemplified by several noteworthy cases of well-known plants that possess distinct canonical scientific names across taxonomic databases. Take kiwifruit as an example, which is identified as *Actinidia chinensis deliciosa* in Catalogue of Life (CoL), *Actinidia chinensis var. hispida* in Encyclopedia of Life (EoL), and *Actinidia deliciosa* in NCBI taxonomy. A similar pattern of inconsistency is evident in cases like wild soybean (designated as *Glycine soja* in NCBI taxonomy) and bitter almond (designated as *Prunus dulcis var. amara* in NCBI taxonomy). Moreover, a considerable amount of the information, especially derived from ethnobotanical literature and traditional medicinal sources, pertains to species identified by vernacular names or alternative non-conventional designations. These names frequently manifest as Latin-inflected variations of canonical scientific names, creating challenges in aligning them with taxonomic references [11, 17]. Another challenge arises from the presence of typographical errors in scientific names, which can originate from misspellings in the original text or errors during digital transposition [18]. In addition to the inherent challenges related to re-formatting chemical structural identifiers, complications arising from scientific names present a significant obstacle to standardizing and integrating NP occurrence information into a comprehensive database.

To address this challenges, algorithmic approaches such as Taxamatch [19] and gnparser [20] have been developed to facilitate the process of recognizing, correcting, and managing scientific names. Taxamatch incorporates a modified Damerau-Levenshtein distance and phonetic algorithm to detect errors within scientific names, thus functioning as a high-throughput scientific name processing algorithm. On the other hand, gnparser identifies semantic elements within scientific names such as abbreviations and authorship, and then processes them to provide the canonical form of the given input. These applications can be utilized to handle tasks related to scientific names, such as curating biodiversity information, thereby facilitating research that would otherwise require significant time to manually curate the data. Unfortunately, current scientific name mapping applications frequently have constraints, including the need for a specific reference file and the inability to effectively correct certain input typos. Given the growing need for smooth and real-time scientific name mapping, there is a clear requirement for more advanced and flexible solutions capable of handling multiple references and effectively rectifying input errors.

Here, we would like to introduce PhyloSophos, a high-throughput scientific name processor designed to provide connections between scientific name inputs and a specific taxonomic system. PhyloSophos is conceptually a mapper that returns the corresponding taxon identifier from a reference of choice: to maximize performance, PhyloSophos can refer to multiple available references to search for synonyms and recursively map them into a chosen reference. It also corrects common Latin variants and vernacular names, which often appear in ethnobotanical literature and natural product research, subsequently returns proper scientific names and its corresponding taxon identifiers. We would like to provide a case-study which demonstrate mapping of scientific names found in four NP occurrence databases, thereby represent the capacity of PhyloSophos to process scientific name variants with superior performance and further emphasize the potential of revitalizing similar biological data resources.

## Results

### Assessment of discrepancies between taxonomic reference databases

**Fig. 1:**
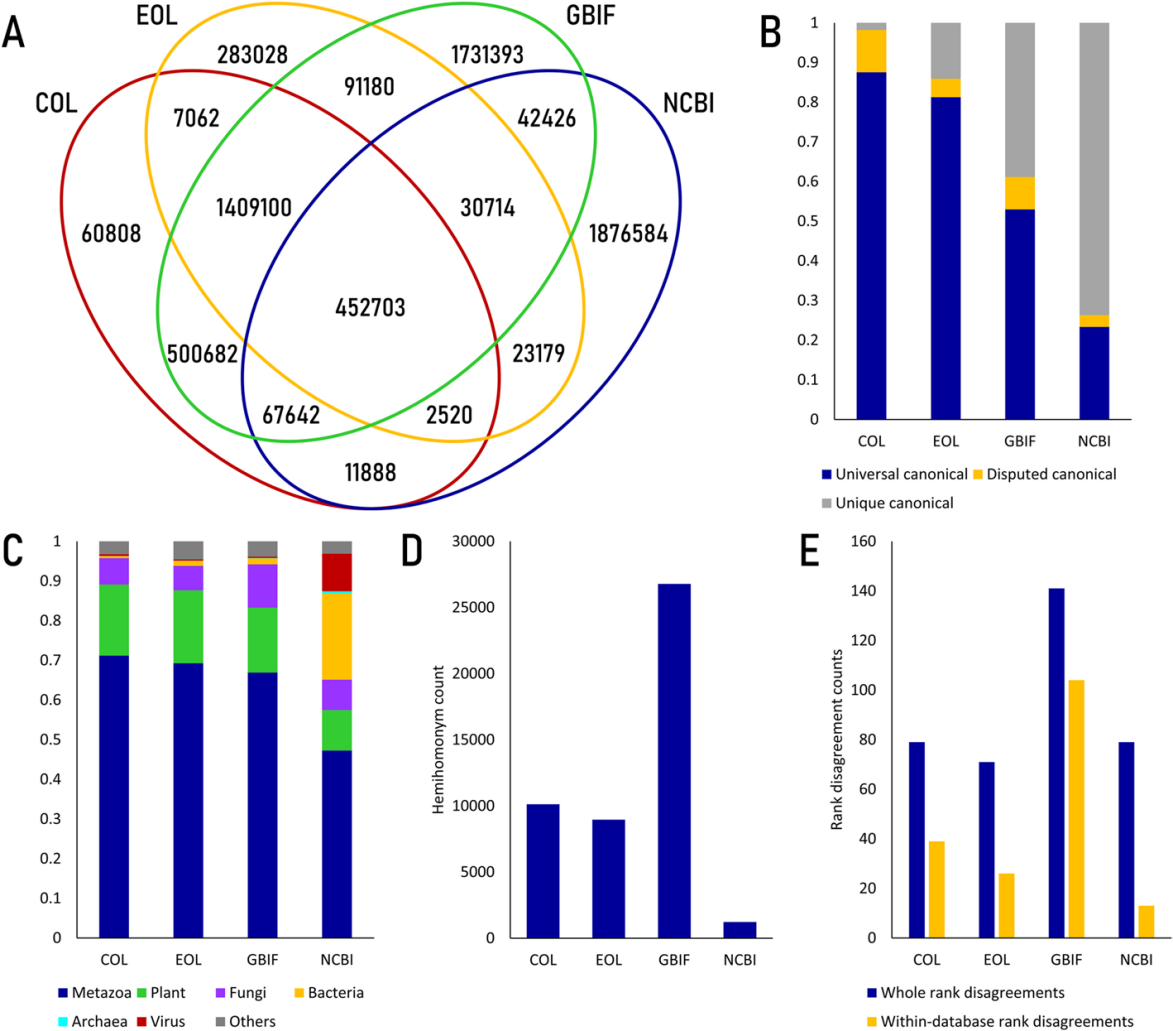
Discrepancies between taxonomic databases. A: Occurrence of canonical scientific names found in four taxonomic reference databases. B: Canonical name-synonym discrepancy. Dark blue: universal canonical names – scientific names which appear only as canonical names in at least one other taxonomic reference database. Gold: disputed canonical names – scientific names appear as synonym of other canonical name in at least one other taxonomic reference database. Grey: unique canonical names – scientific names only appear in a given database. C: Taxonomic composition of entities found within databases. Dark blue: metazoa (animal), Green: plant, Purple: fungi, Gold: bacteria, Cyan: archaea, Dark red: virus, Grey: others. D: Number of hemihomonyms found within taxonomic databases. E: Number of canonical scientific hemihomonyms which correspond to more than two different taxonomic entities with different taxonomic rank. Dark blue: number of whole rank disagreements, Gold: number of rank disagreements found within a single database

In our investigation, we thoroughly examined four taxonomic reference databases—Catalogue of Life (CoL), Encyclopedia of Life (EoL), GBIF, and NCBI taxonomy [21–24]—which led us to unveil significant disparities in the canonical scientific name repertoires among them. Out of the 6.59 million scientific names we analyzed, a mere 450,000 names were found to be common across all four databases, while a substantial 3.95 million names appeared exclusively in one of the four reference databases (Fig. 1A). Intriguingly, CoL-EoL-GBIF shared a substantial number of entries, with 1,409,100 out of 4,713,785 names overlapping, while NCBI taxonomy-specific entries accounted for just under 75%, with 1,876,584 out of 2,507,116 names being unique to NCBI taxonomy (Fig. 1A, B). This finding underscores the fundamental differences in the concepts and approaches employed by each database: while CoL, EoL, and GBIF starts as biodiversity databases providing open-access biodiversity data, NCBI taxonomy is designed as a component of broader NCBI framework, focusing on taxonomic information related to genetic and molecular data. These differences are reflected in the apparent variations in the composition of taxonomic entities found in the databases (Fig. 1C, Additional file 1: Table ST3). Each database exhibited unique specialties; for example, GBIF featured the largest amount of metazoan (animal) entries, while NCBI taxonomy contained a wealth of bacterial and viral entries. This can be attributed to the unique feature of NCBI taxonomy, which accepts strain entities described with genome sequences, 16s rRNA sequences, or other similar information [24, 25], while other databases follow more traditional approaches.

A notable aspect we encountered in the taxonomic databases is the presence of cases where a scientific name is considered a canonical name in one database but is listed as a synonym of another canonical name in a different database. Up to 10% of the canonical names present in one taxonomic database are considered synonyms in another database (Fig. 1B, Additional file 1: Table ST4). Interestingly, these disagreements are not confined to inter-database comparisons but also manifest within a single database: for instance, within GBIF, there are more than 89,000 canonical scientific names listed as synonyms of other taxonomic entities within the same database (Additional file 1: Table ST5). Such disparities can be attributed to the dynamic nature of biological taxonomy, which is inherent and further complicates the scientific name processing: for example, processing scientific names from significantly outdated sources without proper authority citation may lead to multiple errors, underscoring the vital importance of conducting consistent surveillance and implementing regular updates.

Another notable feature we identified during our analysis was the presence of hemihomonyms, which are scientific names applied to multiple different taxonomic groups [26]. Surprisingly, each database contained a minimum of 1000 distinct hemihomonyms (Fig. 1D, Additional file 1: Table ST6). Furthermore, we encountered hundreds of instances where the taxonomic rank of a given scientific name varied across different contexts (Fig. 1E). These discrepancies were observed both within individual databases and across multiple databases (Additional file 1: Table ST7). This could pose a challenge in cases where source names lacked proper specific epithets (e.g., Rubia sp.), introducing an additional layer of complexity to the standardization procedure.

The instances mentioned above can introduce potential ambiguities and underscore the critical importance of accurate taxonomic data integration, thereby emphasizing the need for heightened vigilance when processing biological data that includes scientific name information. This fundamental drive prompted us to develop PhyloSophos, an advanced tool capable of referring to multiple taxonomic references and other syntax information, with the aim of achieving better processing and ultimately enhancing the quality and robustness of research findings in the fields of bioinformatics and systems biology.

### Assessment of the impact of PhyloSophos' core concepts on scientific name mapping

We have systematically evaluated the effects of its individual features through a series of carefully designed case studies, aimed at straightforwardly showcasing their respective advantages.

**Scientific name correction** We evaluated the scientific name correction capabilities of PhyloSophos using lists of scientific names intentionally modified with random typographical errors. PhyloSophos achieved an accuracy rate of 99.87% in mapping scientific names characterized by a single typo, the most prevalent error type encountered in publicly available datasets (Table 1). Out of the 13 name strings that did not align with the original ID, 7 instances failed mapping due to diverse reasons, 4 yielded partial mapping outcomes at the genus level, and 2 were confirmed instances of multiple mapping attributable to two highly similar scientific names.

**Table 1:** PhyloSophos mapping performance for scientific names with typographical errors

Number of typos	Correct match	Incorrect match	Mapping accuracy
0	10,000	0	1.0
1	9,987	13	0.9987
2	9,963	37	0.9963
3	9,956	44	0.9956

Likewise, the mapping accuracy values for the lists containing scientific names with two and three typographical errors stood at 99.63% and 99.56% respectively, underscoring the robustness of its performance across different degrees of typographical complexity.

**Multiple database usage** We conducted a case study utilizing 453,779 name inputs (Additional file 6: Data SD5) that were absent from the NCBI taxonomy but were uniquely acknowledged as canonical scientific names within either CoL, EoL, or GBIF. Our aim was to determine the extent to which these name inputs could be successfully connected to taxa within the NCBI taxonomy using PhyloSophos.

Out of the total 453,779 name inputs analyzed, a subset of 5,434 (1.20%) were successfully linked to species-level taxa (Table 2). Within this subset, 5,418 name inputs were identified as having unique corresponding taxon IDs, with only 16 instances linked to multiple taxa. Among these, 1397 name inputs were categorized as not initially present in the NCBI taxonomy due to minor issues, such as character signs or punctuation. These inputs were aligned with the appropriate taxon through the standard input processing procedure implemented by PhyloSophos. The remaining 4021 name inputs were successfully connected to species-level taxa, primarily leveraging canonical name-synonym relationships and phylogenetic lineage information attainable through the utilization of multiple references. The predominant share of name inputs (n = 437,364, 96.4%) was mapped to taxa at higher taxonomic ranks. The most frequent rank among the associated taxa was the genus (n = 268,594, 59.2%), which could often be assigned a corresponding taxon ID by extracting the first word-block of the name input and matching it to a single taxonomic reference. However, the remaining name inputs (n = 168,770, 37.2%) received taxon IDs at the family level or higher. This particular outcome could only be attributed to the utilization of multiple references and the phylogenetic information they contain. Besides, a small subset of seven name inputs resulted in exceptions, while in the case of 10,974 name inputs (2.42%), the corresponding taxon could not be identified within the NCBI taxonomy.

**Table 2:** PhyloSophos mapping status for canonical scientific names (n = 453,779) uniquely appearing in either Catalogue of Life (CoL, n = 33,639), Encyclopedia of Life (EoL, n = 3,399) and GBIF (n = 416,741) using NCBI taxonomy as a reference system

Scientific name mapping status	Total (n = 453,779)	CoL (n = 33,639)	EoL (n = 3,399)	GBIF (n = 416,741)
Exact mapping	5,434 (1.20%)	613 (1.82%)	905 (26.62%)	3,916 (0.94%)
(raw & simple correction)	1,397	66	21	1,310
(recursive mapping)	4,021	543	881	2,597
(multiple taxa linked)	16	4	3	9
Nearest mapping	437,364 (96.3%)	32,796 (97.5%)	2,452 (72.1%)	402,116 (96.5%)
(genus level)	268,594	21,063	1,658	245,873
(higher taxonomic level)	168,770	11,733	794	156,243
Exceptions	7	0	4	3
Unmapped	10,974	230	38	10,706
Theoretical maximum for single DB usage	269,994 (59.5%)	21,129 (62.8%)	1,682 (49.5%)	247,183 (59.3%)
PhyloSophos mapping	442,798 (97.6%)	33,409 (99.3%)	3,357 (98.8%)	406,032 (97.4%)

**Fig. 2:**
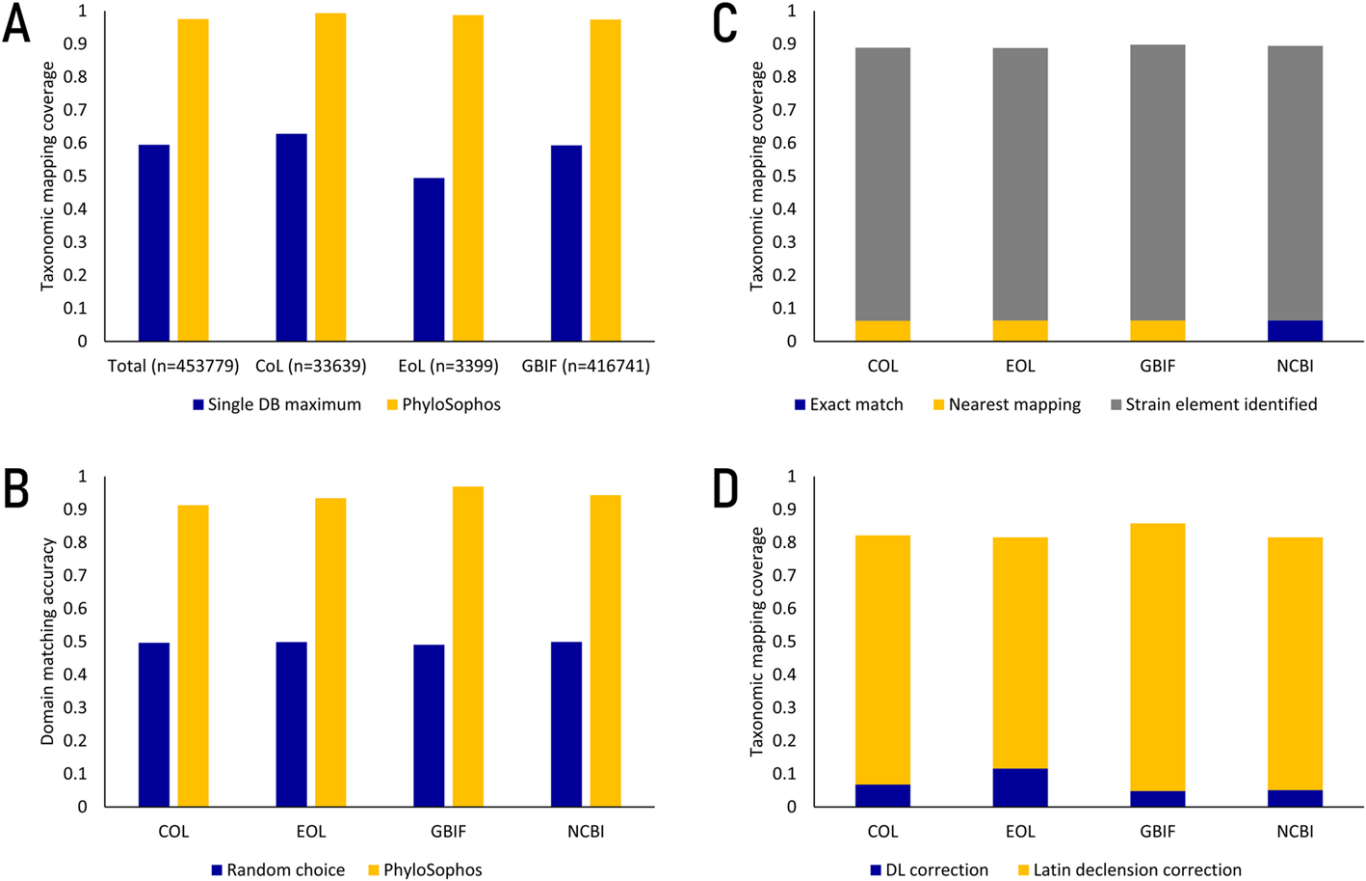
Assessment of the effects of PhyloSophos' core concepts on scientific name mapping. A. Partial mapping coverage could be improved with multiple database usage. (Whole): all canonical scientific names uniquely appear in either CoL, EoL or GBIF. CoL: canonical scientific names uniquely appear in CoL. EoL: canonical scientific names uniquely appear in EoL. GBIF: canonical scientific names uniquely appear in GBIF. Dark blue: Theoretical maximum mapping coverage achievable with single taxonomic database usage. Gold: Mapping coverage achieved with multiple database usage. B. Phylogenetic domain matching accuracy of scientific names with homonymic generic epithets. Dark blue: random choice (null hypothesis). Gold: PhyloSophos mapping result. C. Identification of name inputs with strain-like elements (n = 2,988). Fractions of name inputs which assigned mapping status codes of either 0–5 (exact match code) or 40 (strain name code) were calculated per each taxonomic reference. Dark blue: exact match. Gold: nearest match. Grey: strain-like element identified. D. Reconstruction accuracy of name inputs with Latin declension (n = 353). Fractions of name inputs which assigned mapping status codes 30/31 were calculated per each taxonomic reference. Dark blue: Fraction of name inputs mapped with edit distance (Damerau-Levenshtein) based correction. Gold: Fraction of name inputs mapped Latin declension correction

The count of name inputs that received at least partial mapping to a taxon has risen from 269,994 (59.5%)—which represents the theoretical maximum attainable using NCBI taxonomy as the sole reference—to 442,798 (97.6%) when leveraging four distinct references (Fig. 2A). This observation underscores the effectiveness of PhyloSophos' approach, which harnesses the power of cross-referencing multiple taxonomic references to enhance mapping coverage significantly.

**Phylogenetic lineage usage** As previously discussed in the preceding section, the presence of hemihomonyms within taxonomic databases is a notable concern. Specifically, when conducting scientific name mapping for a name commencing with a homonymic generic epithet, a dilemma arises: if the name does not precisely align with a taxon in a specific taxonomic reference and proceeds to partial mapping, the question arises as to which of the multiple corresponding genera should be connected. This predicament becomes more complex when considering that hemihomonyms often stem from differences in taxonomic authority across distinct domains of life (e.g., Animal, Plant, Bacteria).

To address these intricacies, PhyloSophos is purposefully designed to incorporate phylogenetic lineage information the nearest mapping process, ensuring that the resulting taxa are closely aligned with the original species and do not inadvertently lead to taxa in different domains of life. To demonstrate its performance, first we have identified 8,888 homonymic generic epithets which are associated with multiple taxa, and following 282,008 species-level canonical scientific names starting with one of those generic epithets across four taxonomic references (Additional file 7: Data SD6). PhyloSophos analysis of those name inputs identified substantial amount of name inputs are mapped to genus-level taxa, where the uncertainties arise from the homonymic generic epithets (Table 3). Within these cases, we observed a noteworthy trend: in approximately 95% of instances, PhyloSophos consistently recommended genus-level taxa that shared higher-level taxonomic affiliations, rather than randomly selecting one from the available options (Fig. 2B, Table 4). This strategic approach leads to more phylogenetically accurate mapping outcomes when the exact matching of a scientific name within a taxonomic reference is unattainable. This stands in stark contrast to simple word-block-based mapping, which fails to consider any information regarding the taxon's phylogenetic lineage.

**Table 3:** PhyloSophos mapping status for species-level scientific names (n = 282,008) with homonymic generic epithets collected from four taxonomic reference databases

Scientific name mapping status	COL	EOL	GBIF	NCBI
Exact mapping	184,895	168,936	223,194	101,515
(raw & simple correction)	183,541	162,960	221,540	97,228
(recursive mapping)	485	5,593	64	4,235
(multiple taxa linked)	860	374	1,580	49
Nearest mapping	91,035	106,936	54,145	175,311
(genus level)	88,204	101,426	53,095	158,474
(higher taxonomic level)	2,831	5,510	1,050	16,837
Partial mapping for strain names	4,454	4,455	4,454	4,522
Exceptions	128	126	133	141
Unmapped	1,496	1,555	82	519

**Table 4:** PhyloSophos mapping accuracy of genus-level nearest mapping for species-level scientific names (n = 282,008) with homonymic generic epithets

Scientific name mapping status	COL	EOL	GBIF	NCBI
Total analyzed targets	86,693	100,212	52,945	157,214
(accurate domain mapping)	79,145	93,612	51,337	148,347
(wrong domain mapping)	7,548	6,600	1,608	8,867
PhyloSophos accuracy	0.913	0.934	0.970	0.944
Random choice accuracy (null hypothesis)	0.497	0.499	0.491	0.500

**Recognition of strain-like names** Strain codes frequently exhibit high similarity to each other in terms of their string representation, but they do not inherently convey the true phylogenetic relationships between individual strains. Consequently, employing edit distance-based correction approaches may lead to the suggestion of scientific names that incorporate strain information, resulting in string-wise similarity but lacking accuracy from a taxonomic standpoint. To mitigate this issue, PhyloSophos has been designed to proactively exclude name strings that resemble strain-level scientific names during the mapping process.

As a case study, we examined a set of 2,988 scientific names that incorporated elements akin to strain-level names present in the COCONUT and NPASS databases (Additional file 8: Data SD7). Within this subset, merely 190 name inputs could be exactly matched with their corresponding taxa within the references, leaving the remaining 2,798 name inputs for further processing (Fig. 2C). Instead of opting for edit distance-based correction, PhyloSophos adeptly identified nearly 2,500 of these name inputs as 'strain-like' (mapped with code 40) and subsequently executed a nearest mapping procedure. This strategic approach aimed to provide results that are potentially more scientifically accurate in these instances.

**Latin declension correction** A substantial volume of information regarding natural products finds its origins in databases associated with more 'traditional' sources. Within these sources, species names are frequently presented in the form of vernacular names or alternative non-conventional designations. It is common for these names to appear as Latin-inflected variations of canonical scientific names, which in turn pose challenges when attempting to align them with taxonomic references. To address this issue, PhyloSophos incorporates a dedicated module that reconstructs the canonical nominative case from these inflected word-blocks. This reconstruction process empowers users to map these inputs to their corresponding taxon IDs within the references with greater accuracy and precision.

To demonstrate this capability, we gathered a set of 353 name inputs from the COCONUT and NPASS databases (Additional file 9: Data SD8). These name inputs contained word-blocks commonly employed in the descriptions of materia medica, such as 'herba,' 'radix,' or 'cortex.' These terms do not typically appear in taxonomic databases, and the edit distances between such name inputs and the original scientific names are often substantial, leading to potential errors in the mapping results. However, PhyloSophos adeptly recognized these distinctive word-blocks within the name inputs and successfully reconstructed approximately 75% of the original forms (Fig. 2D). Subsequently, it mapped these reconstructed names to their corresponding taxon IDs, with less than 50 name inputs remaining unmapped.

### The impacts of taxonomic reference(s) selection and morphological consideration on the mappability of scientific names found in NP databases

To investigate the practical impact of taxonomic database differences on scientific name standardization performance, we conducted an analysis of four NP occurrence databases—COCONUT [27], IMPPAT [28], LOTUS initiative [11] & NPASS [29]—to assess scientific name mappability. Our survey revealed an extensive collection of species-compound pair information, totaling more than a million entries (Additional file 1: Table ST2). Although most of these names followed rules of scientific nomenclature, we also identified a significant number of entries containing transcription errors and artifacts, which could lead to misinterpretations and hinder seamless data integration.

**Fig. 3:**
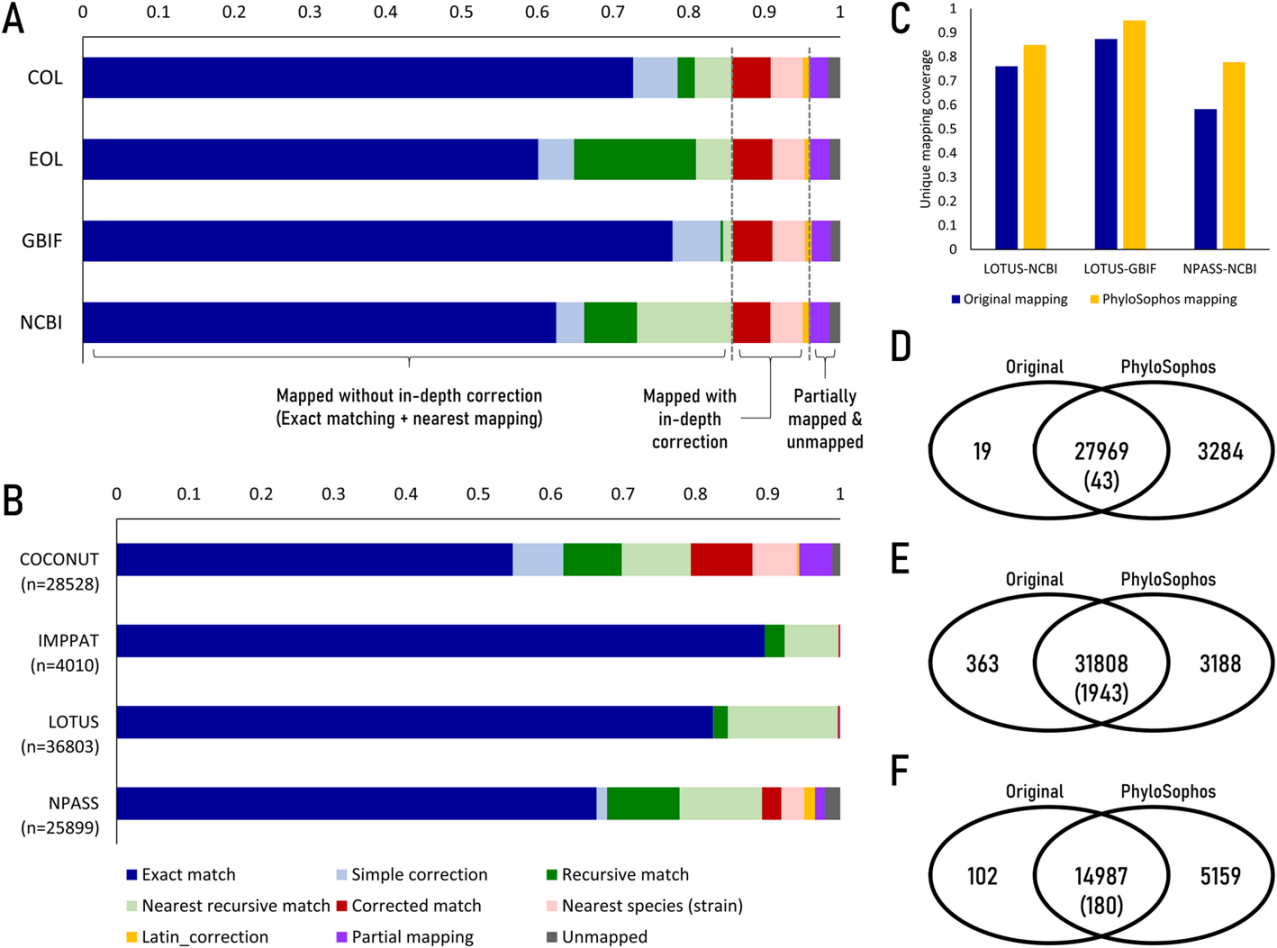
Mapping statistics of scientific names using PhyloSophos. A-B: Raw statistics of scientific name mapping. Dark blue: Exact match, Light blue: Match with simple correction, Dark green: Recursive mapping using other taxonomic DB, Light green: Nearest mapping using other taxonomic DB, Dark red: Mapping with Damerau-Levenshtein correction, Light red: Nearest mapping for strain-level scientific names, Gold: Mapping with Latin declension correction, Purple: Partial mapping, Grey: Unmapped (also see Table 5). A: Results of mapping scientific names found within four NP occurrence databases combined (n = 59,570) using different taxonomic references. B: Results of mapping scientific names found within four NP occurrence databases individually, using NCBI taxonomy as a target taxonomic reference. C. Comparative analysis between PhyloSophos mapping results and the taxonomic mapping provided by the original NP occurrence database. X-axis represents the Taxonomic reference-NP database pairs utilized for the analysis, while the Y-axis indicates the percentage of name inputs correctly mapped to a single corresponding taxon ID. Dark blue: Taxonomic mapping provided in DB metadata. Gold: PhyloSophos mapping. D-F. Diagrammatic representation of mapping status. D-F. Diagrammatic representation of mapping status. Original: counts of taxon IDs provided in the original metadata. PhyloSophos: counts of name inputs which uniquely & precisely assigned with taxon IDs by PhyloSophos. D: Mapping of LOTUS species entries to NCBI taxonomy. E: Mapping of LOTUS species entries to GBIF. F: Mapping of NPASS species entries to NCBI taxonomy

In total, we identified 59,570 unique scientific name strings from the four databases (Fig. 3A, Table 5, Additional file 1: Table ST8). The percentage of scientific names directly matching taxonomic reference entries varied significantly, ranging from as low as 60.11% for EoL to as high as 77.87% for GBIF. Simple corrections, such as removing taxonomic abbreviations and typographical marks, slightly increased the percentage to 64.86% for EoL and 84.20% for GBIF. The lower matching rate observed with EoL was due to its relative lack of synonym information (Additional file 1: Table ST1), which could be supplemented by utilizing other taxonomic references. Through the application of the recursive mapping method, we successfully linked 8,384 name strings (14.07%) to corresponding taxonomic entities within EoL, significantly improving the overall matching rate. Consequently, we achieved mapping rates as low as 73.17% (NCBI taxonomy) and as high as 84.53% (GBIF) of name strings to their respective taxonomic entities in the chosen reference database.

**Table 5:** PhyloSophos mapping status for scientific names (n = 59,570) found in four NP occurrence databases

Scientific name mapping status	COL	EOL	GBIF	NCBI
Exactly matched without correction	43,279	35,812	46,385	37,236
Exactly matched with simple correction	3,500	2,823	3,773	2,197
Recursive mapping for scientific names which are exactly matched using other taxonomic references	1,351	9,595	198	4,154
Nearest taxon mapping for scientific names which are exactly matched using other taxonomic references	2,892	2,907	784	7,553
Matched with edit distance-based correction	3,079	3,108	3,106	2,943
Nearest mapping for strains & species affinis	2,529	2,523	2,553	2,531
Latin declension correction	490	439	527	480
Partial mapping	1,494	1,516	1,494	1,615
Unmapped	956	847	750	861

The relatively lower matching rate observed with NCBI taxonomy can be attributed to its relative lack of species-level taxonomic entities within the plant and fungal categories (Fig. 1C), which are where the majority of reported NPs originate (Fig. 3B). Our approach, PhyloSophos, avoids applying an in-depth correction algorithm to name strings that already match a specific taxonomic entity in at least one reference database. Instead, we focus on identifying the lowest possible taxonomic rank within the database that encompasses the taxon, thereby preventing the erroneous application of the correction algorithm to scientifically valid names. By adopting this approach, an additional 7,553 name strings were successfully assigned to the nearest possible taxonomic entity within NCBI taxonomy, significantly increasing the mapping rate to 85.84% overall.

During the additional inspection process, the remaining 8430 (14.16%) name strings underwent scrutiny to identify possible typos and other relevant issues, leading to the discovery of three distinct problems. First, approximately 3000 name strings contained typos in either the specific epithet or generic epithet, which were effectively detected and corrected using the Damerau-Levenshtein distance-based typo correction algorithm. Second, about 2500 name strings included a taxonomic glossary denoting the association with a specific strain within a species. As these strain IDs were typically composed of a combination of alphabets and numbers without a specific linguistic morphological structure, applying a typo correction algorithm to these names would have resulted in significant mapping errors. To address this, we opted to assign the nearest species- or genus-level taxonomic entities that included the particular strain. Lastly, approximately 500 name strings exhibited Latin declension problems, which could have occurred as either typos or intrinsic errors originating from the primary data source. Overall, the mapping process left fewer than 1000 names, a significant improvement compared to the possibility of leaving up to 23,758 names unmapped (depending on the utilized database), allowing the vast majority of species-compound pair information to be salvaged and contributing to the robustness and reliability of our study's findings.

We tried to map the scientific name strings from each NP occurrence database to the corresponding taxonomic entities in NCBI taxonomy (Fig. 3B, Table 6, Additional file 1: Table ST9). The name strings found in IMPPAT and LOTUS databases were successfully mapped to a nearest taxonomic entity within NCBI taxonomy with an impressive mapping rate of over 99% without in-depth correction process. In contrast, a significant number of name strings from COCONUT and NPASS databases required a more thorough correction process, resulting in up to 2.1% of names being left unmapped. The majority of name strings with Latin declension problems were identified in NPASS, which likely originated from collecting NP occurrence information from traditional medicine-themed databases [30].

**Table 6:** PhyloSophos mapping status for scientific names found in in the individual NP occurrence databases using NCBI taxonomy as a reference system

Scientific name mapping status	COCONUT	IMPPAT	LOTUS	NPASS
Exactly matched without correction	15,617	3,591	30,338	17,178
Exactly matched with simple correction	1,994	0	13	376
Recursive mapping for scientific names which are exactly matched using other taxonomic references	2,304	111	735	2,595
Nearest taxon mapping for scientific names which are exactly matched using other taxonomic references	2,724	299	5,597	2,950
Matched with edit distance-based correction	2,432	8	74	694
Nearest mapping for strains & species affinis	1,750	0	0	814
Latin declension correction	96	0	0	390
Partial mapping	1,296	1	38	349
Unmapped	315	0	8	553
(Total)	28,528	4,010	36,803	25,899

To assess the performance of PhyloSophos, we conducted a comparative analysis using taxon IDs provided in the NP database raw metadata. LOTUS provides NCBI taxonomy IDs and GBIF taxon IDs for 27,988 (76.0%) and 32,171 (87.4%) species entries respectively, while NPASS provides NCBI taxonomy IDs for 15,089 (58.3%) species entries (Fig. 3C). PhyloSophos significantly improved the mapping coverage to 84.9% for LOTUS-NCBI mapping, 95.1% for LOTUS-GBIF mapping, and 77.8% for NPASS-NCBI mapping. Upon further examination of LOTUS-NCBI mapping, we discovered that out of 36,803 unique scientific name strings, 27,969 names were mapped in both datasets, while 3,284 names were exclusively mapped in our PhyloSophos results. Only 19 names were uniquely mapped in the raw metadata (Fig. 3D). A closer look at these 19 names revealed two main reasons why PhyloSophos failed to provide mapping information. First, some scientific names were updated in the NCBI taxonomy, and our mapping algorithm could not accurately follow these updates at the species level resolution. Second, we found cases where the original name strings consisted of single-word generic epithets, referring to genera that also had subgenera with the same name (Additional file 1: Table ST10). Additionally, we identified 43 instances of disagreement between the PhyloSophos mapping results and the NCBI IDs provided in the original metadata. Through individual manual curation, we determined that most of these disagreements arose due to periodic updates of taxonomic references (Additional file 1: Table ST11).

On the other hand, when comparing the GBIF IDs found in LOTUS metadata with our PhyloSophos results, a significantly large number of scientific names (n = 1943) exhibited identifier disagreements (Fig. 3E). Upon careful investigation of these disagreement cases, we discovered that virtually every GBIF ID in the LOTUS metadata pointed to taxonomic entries that are now considered non-canonical (e.g., Synonyms, Homonyms, Doubtful taxa, etc.), which need to be superseded by the IDs identified by PhyloSophos to ensure accurate and up-to-date taxonomic information (Additional file 1: Table ST12).

Analysis using NPASS species entries and NCBI taxonomy ID mapping information resulted in a largely analogous pattern to that observed in LOTUS-NCBI mapping (Fig. 3F), suggesting similar underlying reasons for the observed discrepancies. These findings underscore the robustness of PhyloSophos' comprehensive taxonomic mapping approach, which can be employed for improved scientific name standardization and mapping against dynamically changing taxonomic databases.

### Integration of multiple NP occurrence databases using standardized workflow

**Fig. 4:**
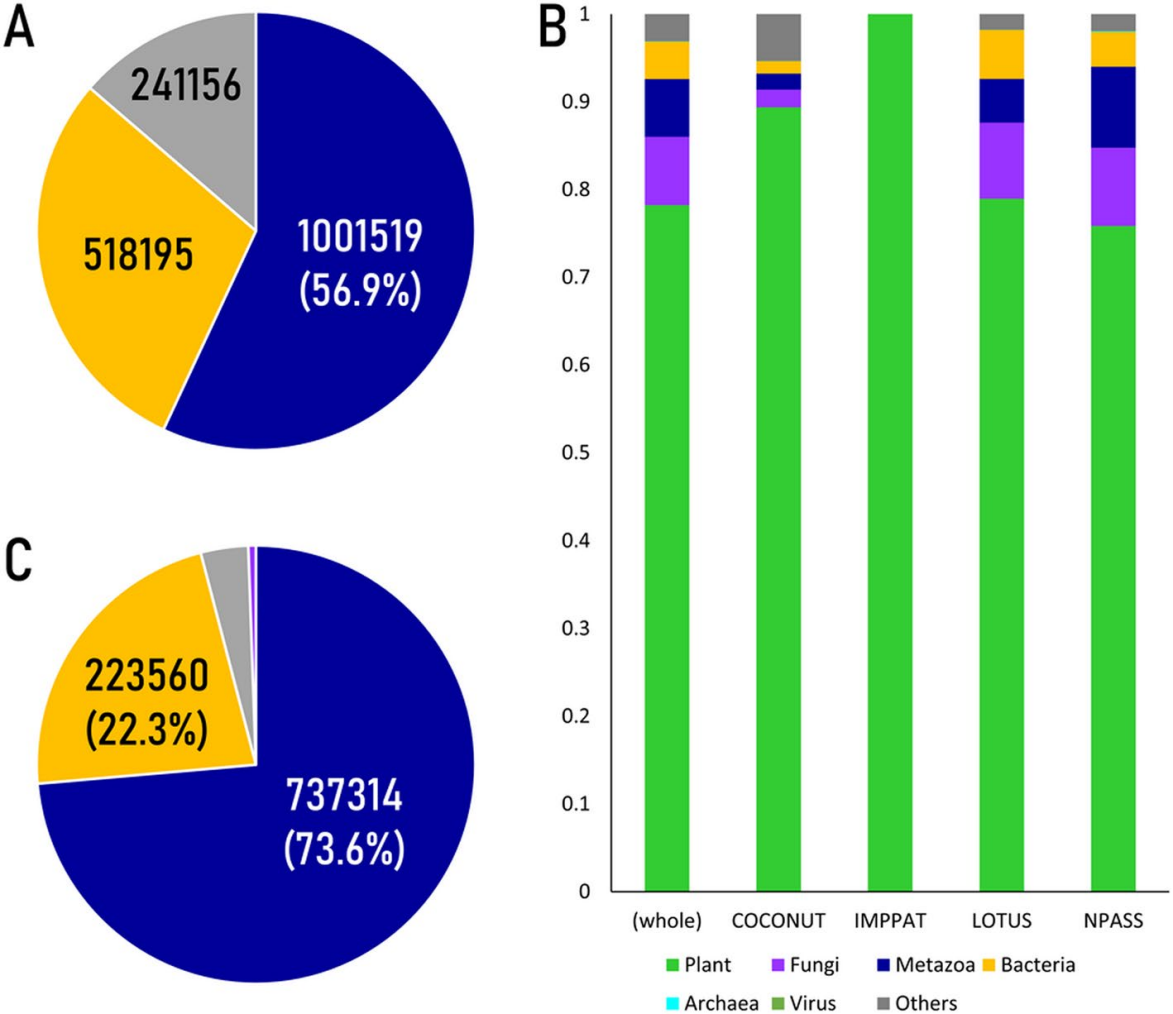
Integration of species-compound pair information using standardization pipeline. A: Statistics of species-compound pairs collected from four NP occurrence databases. Dark blue: unique species-compound pairs, Gold: duplicate species-compound pairs (denote multiple support from NP occurrence databases), Grey: pairs which contain scientific names that are not mapped to NCBI taxonomy at least at species level. B: Taxonomic composition of scientific names found in species-compound pair information. Green: plant, Purple: fungi, Dark blue: metazoa (animal), Gold: bacteria, Cyan: archaea, Dark red: virus, Grey: others. C: Degree of support of species-compound pairs. Dark blue: one database (n = 737,314), Gold: two databases (n = 223,560), Grey: three databases (n = 35.046), Purple: four databases (n = 5,609)

Using the scientific name mapping information, we embarked on compiling an integrated dataset from the species-compound pair information retrieved from the four NP occurrence databases. Out of the 1,760,870 pairs of information found in the databases, we successfully associated 1,519,714 pairs with proper reference IDs (Fig. 4A). After further processing, we were left with 1,001,529 unique species-compound pairs, consist of 35,767 different species and 175,471 different metabolites. Remarkably, the size of this dataset is almost 1.5 times as large as the largest data compendium used, the NPASS, implying its potential for incorporating more databases and expanding even further.

In our quest for a comprehensive understanding of the distribution and prevalence of unique species-compound pairs, we embarked on an in-depth analysis of their sources and origins. Within this dataset, plants emerged as the primary contributors, and we also identified additional fungi and animals among these associations (Fig. 4B, Additional file 1: ST13). Notably, we observed a clear distinction between IMPPAT, with its exclusive focus on medical plants [28], and COCONUT/LOTUS, a more general database [11, 27], in terms of taxonomic group representation. This variability underscores the importance of considering database-specific biases and preferences when utilizing NP occurrence data for research and analysis.

Moreover, the relatively high number of unique pairs compared to the total used pairs highlights an intriguing aspect of our findings (Fig. 4C). The vast majority of these pairs are supported by evidence from only a single database, with 737,314 pairs appearing in a single NP occurrence database and only 5,609 pairs enjoying support from all four databases. This observation draws attention to the diversity and heterogeneity of information available in the NP occurrence databases. Notably, a notable association with plant species was observed among the well-supported pairs, while the support for fungal / animal / bacterial metabolites was comparatively lower (Additional file 1: Table ST14). This discrepancy accentuates the need for further exploration and cross-validation to fully comprehend the intricate relationships and unique characteristics inherent in NP occurrence data. Overall, these valuable insights provide a foundation for advancing our understanding of the vast possibilities and complexities within the realm of natural product research.

## Discussion

Scientific names have played a crucial role in taxonomy and biological research, facilitating effective communication among researchers over a long history [31]. Nevertheless, it is crucial to acknowledge that the application of scientific names within the context of the information era presents certain challenges and limitations, which may stem from various factors, including the traditional structure of scientific names, the governance system, and the real-world usage practices surrounding them [10, 31, 32]. These factors, compounded by researchers' indifference to embrace the digital transformation of taxonomic information, have impeded the systematic research necessary to tackle challenges associated with scientific names. For instance, the absence of a 'gold standard' dataset for evaluating scientific name mapping models, a task that requires the expertise of taxonomy experts who are seldom inclined toward information processing, presents a significant hurdle to the development of accurate models. To circumvent this obstacle, we resorted to creating testing sets containing name strings with randomly introduced typographical errors or manually collected examples of erroneous scientific names, subsequently evaluating model performance based on these datasets.

This issue becomes more pronounced when considering NP-related data, as there has been historically limited incentive to regularly update and integrate it into a comprehensive system [15, 33]. The decentralized nature of data collection and dissemination in this field makes periodic updating and integration into a unified system challenging, leading to fragmented, incomplete, and unstandardized NP-related data, hindering its effective use and integration with other scientific resources. To effectively address this challenge, researchers from diverse backgrounds must coordinate efforts to incentivize the regular updating and streamline the integration of natural product data into a unified framework, ensuring its accessibility for researchers in various domains. One of the challenges that could not be resolved by information-based biologists relates to the inherent discrepancies observed in taxonomic references, which emerge as a result of the diverse perspectives and interpretations held by individual taxonomists [34]. At the same time, continuous accumulation of high-throughput evidence, driven by technological advancements, further compounds the complexity of the issue: the influx of data often presents conflicting phylogenetic evidences regarding the interrelationships among taxa, necessitating rearrangements within taxonomic systems to accommodate new discoveries [6, 31]. The ongoing nature of this challenge highlights the intricate and ever-evolving nature of taxonomy, necessitating the expertise of dedicated taxonomists to address and resolve these discrepancies.

The work presented can be regarded as both a dedicated effort and a pragmatic compromise aimed at providing an optimal solution within the intricacies of the aforementioned situation. Recognizing that it is inappropriate to arbitrarily designate a single taxonomic reference as the exclusive authority over others, we have developed a workflow that considers multiple available taxonomic systems, enabling us to accurately determine the taxonomic identity of a given input and find the best match within the system of choice. Similarly, we have incorporated a Latin declension correction algorithm and a manually curated name list to augment the resolving power required for deciphering heavily modified species names. By embracing this approach, PhyloSophos could handle several problems which other tools such as Taxamatch [19] could not: processing of sub-specific scientific names, providing phylogenetically accurate nearest taxon mapping, correcting systematically modified names, and offering thoughtful treatment of scientific names with specific syntax elements could be counted among examples. This approach would ensure that PhyloSophos becomes a versatile solution capable of meeting the diverse needs of users, thereby advancing our collective understanding of the subject matter.

The significant disparities observed among NP occurrence databases were quite notable. Only a small proportion of NP-species pairs were found in every assessed database, while the majority of pairs relied on evidence from a single database. This observation actually points out several issues: first, the existence of multiple sources of evidence supporting NP-species pairs strongly suggests the presence of representative metabolites specifically associated with each species. These representative metabolites are distinguished by their consistent occurrence and often exhibit distinct physiological activities. The substantial 'evidence count' linked to these pairs can be deemed an indicator of their relative importance and potential clinical significance, laying the groundwork for further investigation into their biomedical applications. Secondly, primary references containing information on species-compound pairs, which constitute the foundation of natural product data, are marred by significant inadequacies in reporting standards [11, 33]. This issue can be attributed, in part, to the prevailing convention among researchers who primarily contribute to the field. It is noteworthy that a majority of natural product research articles, even the most recent ones, lack machine-readable chemical structural identifiers such as SMILES or InChI, necessitating substantial efforts for manual conversion into digital formats. This presents a formidable challenge for database organizers tasked with curating primary references. Given the vast scale of data, manual verification becomes virtually untenable, leading to potential discrepancies in the information incorporated within each database, contingent upon the specific data processing methodologies employed by each database. Finally, it may be more advantageous to redirect our focus towards frequently occurring metabolite scaffolds and recurring biochemical patterns observed within specific species, rather than solely fixating on individual compounds. This shift in perspective acknowledges not only the aforementioned incompleteness of digitized annotations, but also the fact that various factors, such as seasonal variations, environmental differences, and the presence of external stress, can influence the expression of a diverse array of metabolic enzymes within plant tissues, resulting in distinct metabolite profiles reported in individual research articles [35]. By recognizing these dynamic factors and directing attention towards broader patterns, researchers might gain a deeper understanding of the underlying biological activities that define a particular species' metabolite profile, ultimately advancing our understanding of the vast possibilities within the realm of natural product research.

Despite the fact that there is still room for computational optimization, such as reducing memory usage, PhyloSophos is already capable of delivering reliable results for practical research. We expect this application will help facilitate the systematic incorporation of disparate biological data, such as biodiversity reports and ethnobotanical annotations, into the knowledge network, thereby providing access to high-throughput analysis. The resulting data platform could be utilized in solving several challenging problems in the field of natural product research: for example, it would expedite the identification of natural products from the MS spectra of biological samples, which often require time-consuming experimental measurement and manual curation by expert biochemists. Additionally, the platform would enable efficient dereplication of previously assessed natural products, which is particularly relevant in drug discovery and bioprospecting. Embracing this approach would significantly accelerate the overall analysis and research process, leading to faster and more streamlined investigations in the dynamic field of natural products.

## Conclusion

Through our research, we have successfully demonstrated systematic mapping capabilities of PhyloSophos, enabling precise matching of scientific name inputs to their corresponding taxa within the chosen reference system. Leveraging the principles of taxonomy and nomenclature, we employed various strategies, such as rule-based preprocessing and multiple reference-based mapping, resulting in significant improvements to our application's performance. This, in turn, led to enhanced standardization of natural product (NP) occurrence data from diverse sources, successfully connecting thousands of previously unlinked scientific names to their respective taxonomic entities within the reference, and seamlessly integrating this valuable information into a comprehensive dataset containing over a million unique species-compound pairs.

Overall, PhyloSophos represents a significant advancement in enhancing the accessibility, accuracy, and efficiency of scientific name mapping, thereby driving progress in exploring the diverse and rich realm of natural products. Its versatility and adaptability make it a valuable tool for accelerating data integration, thereby enhancing our research capacity and paving the way for exciting discoveries in the realm of natural product research.

## Methods

### Data sources

As our primary references, we employed four taxonomic databases: Catalogue of Life [21], Encyclopedia of Life [22], GBIF [23], and NCBI taxonomy [24]. To facilitate data analysis, we programmatically downloaded all metadata (version 230720), which was subsequently processed into table files for enhanced readability and ease of use.

We conducted an analysis of four NP occurrence databases of following versions: COCONUT (version January 2022) [27], IMPPAT (version 2.0) [28], LOTUS (version 230,106) [11], and NPASS (version 2023) [29]. Database statistics are shown in Additional file 1: Tables ST1/ST2.

### Reference data preprocessing

**Fig. 5:**
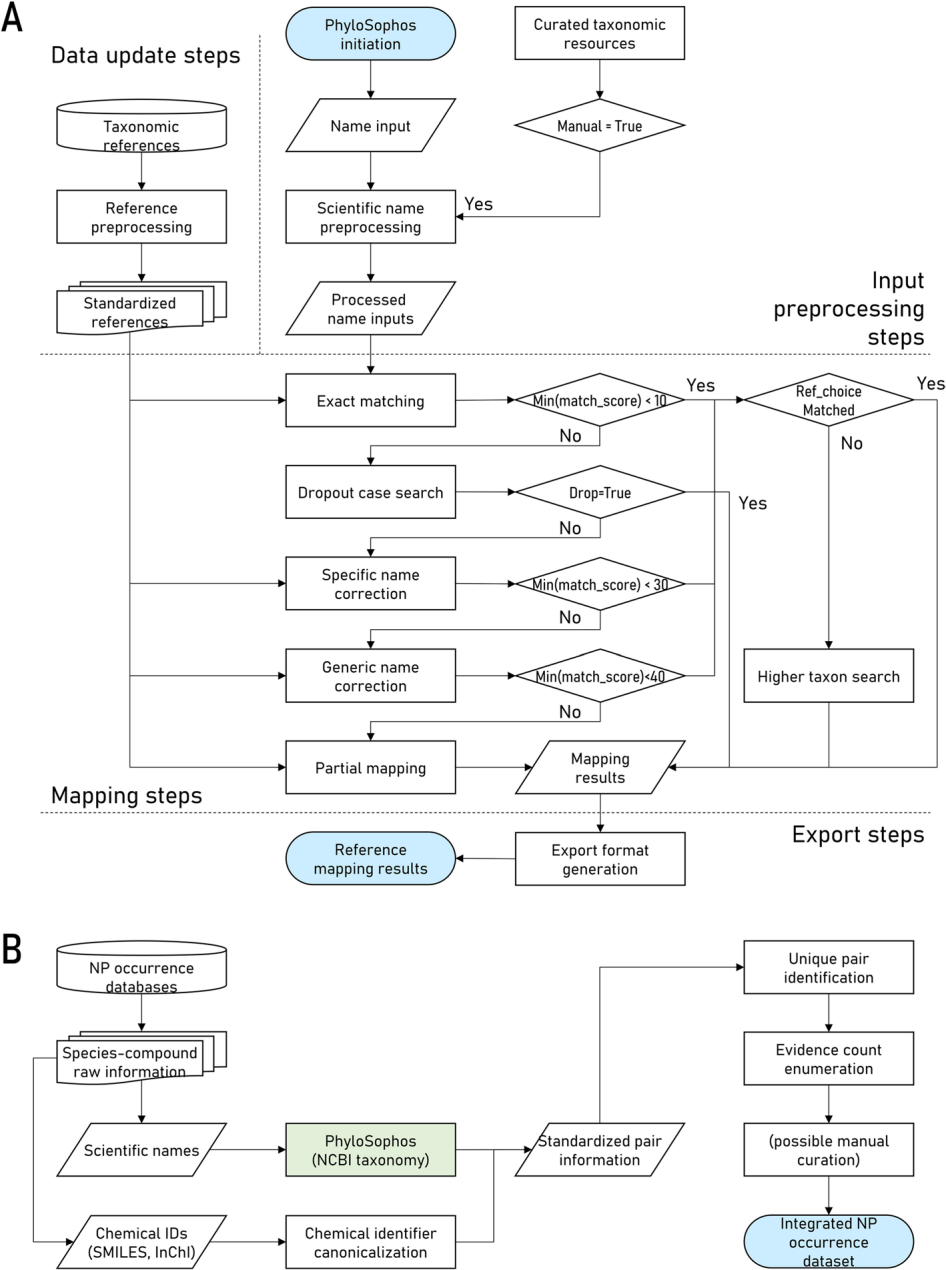
Flowcharts for analysis of scientific names within databases. A: Flowchart of PhyloSophos. B: Flowchart for integration of species-compound pair information found within NP occurrence databases

PhyloSophos is a Python-based standalone package that could be divided into four parts: reference data preprocessing, input name preprocessing, scientific name mapping, and mapping result export (Fig. 5A, Additional file 1: Text S1/S2).

The configuration of the taxonomic references is achieved through the execution of distinct Python update scripts: 'phylosophos_initialize_update.py' for CoL/EoL/NCBI taxonomy and 'gbif_preprocessing.py' for GBIF. These scripts allow users to download and integrate current taxonomic information from the aforementioned reference databases. The utilized reference databases encompass all domains of life, incorporating phylogenetic tree data and comprehensive synonym information, which are publicly accessible via FTP servers. The user may further customize their own PhyloSophos environment by placing additional taxonomic reference files within the reference directory (Additional file 1: Text S4).

Each metadata package comprises core taxonomy files in a tabular format (e.g., CoL: Taxon.tsv, EoL: taxon.tab, GBIF: Taxon.tsv, NCBI taxonomy: names.dmp & nodes.dmp). The update scripts parse these raw files and extract essential information, including (1) database taxon ID, (2) canonical scientific name, (3) existing synonyms, (4) taxonomic rank, (5) phylogenetic lineage, and (6) taxonomic ranks of taxa found within the phylogenetic lineage. This extracted data is then exported as a reference file, denoted as '[reference name]_node_dict.txt,' for each respective taxonomic reference.

Additionally, the update scripts compile the initial word-blocks from both canonical scientific names and synonyms. Subsequently, they enumerate all taxon IDs starting with the given word-block: this information is saved in a reference file named '[reference name]_genus_dict.txt' for each taxonomic reference.

### Input name preprocessing

Input preprocessing, scientific name mapping, and result export are accomplished through the execution of the core PhyloSophos script, 'phylosophos_core.py.'

Files containing scientific name strings, each separated by a newline character, can be imported either as an entire directory (the default setting) or as a specific file. Prior to processing imported inputs, PhyloSophos initiates the importation of all reference files from the '/pp_ref' directory and subsequently generates a comprehensive list of scientific names. This list includes both canonical names and synonyms, encompassing any name recognized as valid within at least one taxonomic reference.

Each raw name string is initially subjected to a case-insensitive match with scientific names from the generated list. This process identifies names that should not undergo further processing. Subsequently, rule-based simple corrections are applied, such as the removal of authority information (e.g., L.) or the elimination of word-blocks enclosed within parentheses. These corrections aim to simplify the given query into its most basic form appearing in taxonomic references.

For queries that do not exactly match any taxonomic names found within the incorporated references, a semantic rule-based screening is executed to determine whether they should be treated differently or excluded prior to the mapping step. The screening process begins by searching for specific taxonomic abbreviations that require special treatment. For instance, if abbreviations like 'cf.' or 'aff.' are detected within a query, indicating similarity to a given taxon but not an exact match, PhyloSophos first removes these abbreviations, attempts to find a match, and then returns the identifier of a higher taxon present within the phylogenetic lineage of the match, thereby reflecting the intended connotation of the abbreviation. Similarly, if a string includes numeric characters unrelated to described years, it is labeled as 'strain-level' and undergoes nearest mapping instead of edit distance-based correction. Additionally, queries containing specific word-blocks such as 'virus,' 'phytoplasma,' or 'endosymbiont' are disregarded since the scientific name within the query, often that of the host species, does not convey taxonomic information about the query itself. Lastly, queries featuring hybrid marks are also flagged and ignored.

The correction of Latin declensions is employed to accurately identify scientific names in their various forms, a common occurrence in traditional medicine-related resources. This process initiates by eliminating word-blocks that are typically not integral components of scientific names, such as terms indicating specific parts of a species (e.g., ‘Herba’, ‘Radix’, ‘Folium’). Next, it generates a list of potential nominative forms according to Latin declension rules from the word-blocks in their genitive case. These potential forms are then combined systematically using the itertools.product module and compared with scientific names present within the reference. This methodology enables users to ascertain the reconstructed canonical form of the provided query. In addition, PhyloSophos can also take a manually curated list of [vernacular name-scientific name] pairs and apply it during the pre-processing step, which allows for further translation of vernacular names before the mapping step and thus expedites the processing.

### Scientific name mapping

The scientific name mapping workflow within PhyloSophos can be described as follows:

- Exact matching: PhyloSophos initiates by attempting to find an exact match with the given input string, either as-is or after applying simple corrections. If an exact match is located in a particular reference, it is assigned a status code ranging from 0 to 5, depending on the exact mapping status for that reference. In cases where a match is found in one or more references but not in others, PhyloSophos collects synonym information from the matched taxonomic entity and conducts a recursive search in the references without an exact match. If a match is discovered through this recursive search, it is assigned a status code of 6 or 8.
- Nearest taxon mapping: When the given input is precisely mapped to a taxonomic entity in at least one taxonomic reference (either to the canonical name or synonym), PhyloSophos recognizes the input as a valid scientific name and does not subject it to edit distance-based correction. Instead, it references phylogenetic lineage information from the previously matched taxonomic entity and endeavors to identify the nearest taxon (with the lowest taxonomic rank) for references lacking an exact match. These mappings are assigned a status code ranging from 10 to 18, contingent upon the taxonomic rank of the provided taxon.
- Rule-based input dropout: Prior to initiating edit distance-based correction methods, PhyloSophos performs a preliminary assessment to determine if the given input contains specific keywords that warrant exclusion from further processing. If the input includes strain names or similarity-associated abbreviations (as identified during the preprocessing step) but cannot be exactly matched with any valid scientific name, PhyloSophos bypasses edit distance-based correction. Instead, it triggers a nearest mapping process, assigning a status code in the range of 40–49. Conversely, if the input contains word-blocks requiring special attention, the subsequent mapping process is halted, and a distinct status code in the range of 90–99 is assigned based on the specific keyword identified within the input.
- Edit distance-based correction (specific epithet only): Edit distance calculations are performed using the Damerau-Levenshtein distance algorithm [36]. As this calculation is computationally intensive, PhyloSophos first searches within a limited pool of scientific names. It initially searches for scientific names sharing the same first word-block, then further narrows down the pool based on the length and character composition of each string. After calculating the edit distance, PhyloSophos returns the scientific name with the lowest edit distance as the corresponding taxonomic entity, accompanied by a status code ranging from 20 to 24, depending on the mapping status.
- Edit distance-based correction (whole input): If mapping remains unattainable with the previous steps, PhyloSophos attempts to correct the input using a similar edit distance-based approach, considering the entire input string. This correction process also includes Latin declension correction and is assigned a status code ranging from 30 to 36, based on the mapping status.
- Partial mapping: In cases where mapping is not achieved through the preceding steps, PhyloSophos attempts to provide partial mapping results using a portion of the given input. If a partial mapping is successful, it is assigned a status code of 100; otherwise, it receives a status code of 1000.

For comprehensive information regarding mapping status codes, please refer to Additional file 1: Text S3.

### Mapping result export

The computed mapping results are aggregated into a dedicated result file for each input file and subsequently exported to the '/result' directory. These result files are named in the format 'phylosophos_result_[export_date]_[export_time]_[input_file_name].txt,' where [export_date] and [export_time] are represented as six-digit numerical values. The identified mapping results are presented in a tabular format, inclusive of their mapping status. Additionally, all other 'best matches' identified in different databases are included in the table. This comprehensive presentation not only provides insights into whether the individual query corresponds to a valid scientific name absent from the chosen reference but also aids in determining if it may not be a scientific name altogether.

### Case studies for PhyloSophos performance evaluation

To demonstrate mapping performances and strong points of PhyloSophos, we have performed the following case studies.

- Core performance evaluation: To quantitatively evaluate the fundamental performance of PhyloSophos in rectifying typographical errors within scientific names, we produced three lists of scientific names, each comprising 10,000 canonical name strings obtained from NCBI taxonomy. These lists were deliberately modified with randomly introduced typographical errors at magnitudes of 1, 2, and 3, respectively. Subsequently, we conducted a comparative analysis by aligning PhyloSophos mapping outcomes with the original NCBI taxonomy IDs, thereby computing the mapping accuracy.
- Analysis of the effects of using multiple taxonomic references: Using the list of canonical scientific names which only appear in a single database, we assessed whether PhyloSophos could utilize canonical name-synonym relationships and phylogenetic lineage information to assign corresponding (or nearest) taxon ID to a name input which do not appear in a given database. We collected 453,779 species-level scientific name inputs which uniquely appear in either CoL, EoL or GBIF, then we assessed how PhyloSophos maps these species name inputs to taxa within NCBI taxonomy. This analysis enabled us to discern the extent to which the name inputs could be linked to species-level taxa through canonical name-synonym relationships and, conversely, how much of the name input was associated with higher taxa (that the scientific name itself could not convey) through phylogenetic lineage information, thereby underscoring the advantages derived from leveraging multiple taxonomic references as opposed to relying on a single reference.
- Analysis of scientific names with homonymic generic epithets: Using the list of scientific names which generic epithet is shared between multiple different taxa, we assessed whether PhyloSophos properly recognizes these inputs and provide accurate phylogenetic lineage. We collected 282,088 species name inputs with generic epithets which correspond to more than two taxa and analyzed it with PhyloSophos. For the name inputs subjected to this analysis, we conducted a comparison between the phylogenetic information derived from the original taxonomic references associated with each species name input and the phylogenetic information acquired through the mapping process. Our objective was to ascertain whether these sources concurred in terms of taxonomic domains (e.g., Animal, Plant, Bacteria). This analysis allowed us to evaluate the accuracy of PhyloSophos' nearest mapping in the context of phylogenetics.
- Strain name recognition test: Using the manually curated list of species names with a clear sign of strain IDs, we assessed whether PhyloSophos recognizes these inputs and waives edit-distance based correction. We collected 2,988 names from COCONUT and NPASS databases (Additional file 2–9: Data SD) and checked whether it assigns correct mapping status of 40, if no exact matching is achieved.
- Latin declension correction test: Using the manually curated list of species names with a clear sign of Latin declension, we assessed whether PhyloSophos recognizes these inputs and correct it into canonical form accordingly. We collected 353 names from COCONUT and NPASS databases (Additional files 2–9: Data SD) and checked whether it assigns correct mapping status of 30/31.
- Comparative analysis of NP database mapping coverage: Using the precalculated species name-taxon ID list provided within LOTUS and NPASS raw metadata, we performed a comparative analysis whether PhyloSophos could associate species names with corresponding taxon IDs with superior accuracy or coverage. LOTUS (n = 36,803) provides taxon ID for GBIF (n = 32,171 / 87.4% coverage) and NCBI taxonomy (n = 27,988 / 76.0% coverage). NPASS (n = 25,899) provides taxon ID for NCBI taxonomy only (n = 15,089 / 58.3% coverage). We assessed mapping coverage to determine how many species name inputs within each NP database could be successfully mapped to unique corresponding taxon IDs by PhyloSophos. Additionally, we systematically documented instances where discrepancies arose between the original taxon ID list and the mapping outcomes produced by PhyloSophos. These cases underwent a manual curation process to ascertain which source more faithfully aligns with the current taxonomic reference.

### Application of PhyloSphos in standardization and integration of NP occurrence databases

We aimed to construct a comprehensive and unified dataset containing NP occurrence information utilizing PhyloSophos (Fig. 5B). To initiate the process, we extracted raw species-compound pair information from the downloaded metadata. Each pair information comprised the correspondence between a scientific name and a chemical structural identifier, along with other database-specific annotations, such as bibliographical references. See Additional file 1: Text S6 for further information.

To ensure precise taxonomic mapping of the identified scientific names, we employed PhyloSophos, which systematically mapped the names to the appropriate taxon found in the NCBI Taxonomy. This strategic selection was motivated by the high compatibility of the NCBI-affiliated databases, such as GenBank and PubChem, potentially enhancing the utility of the resulting dataset. Concurrently, the chemical structural identifiers, whether in SMILES or InChI format, were subjected to systematic conversion to canonical SMILES using RDKit, ensuring uniform application of parameters and rules across all identifiers. Following this, the standardized pairs were merged to create a unified database, providing a standardized representation of NP occurrence data.

### Supplementary Information

**Additional file 1**. Within this supplementary material file, we present comprehensive information regarding PhyloSophos and its mapping performance. The supplementary text section delves into technical aspects of PhyloSophos, elucidating topics such as the interpretation of mapping status codes and the integration of additional reference files. The supplementary figures section offers pseudocodes that serve to enhance comprehension of PhyloSophos' inner workings. Additionally, the supplementary tables section furnishes a compendium of database statistics and analysis outcomes, delineating PhyloSophos' proficiency in recognizing and rectifying real-life scientific name errors.

**Additional file 2**. PhyloSophos analysis results of species entries (n=28,528) from COCONUT database.

**Additional file 3**. PhyloSophos analysis results of species entries (n=4,010) from IMPPAT database.

**Additional file 4**. PhyloSophos analysis results of species entries (n=36,803) from LOTUS database.

**Additional file 5**. PhyloSophos analysis results of species entries (n=25,899) from NPASS database.

**Additional file 6**. List of canonical scientific names which uniquely appear in one of taxonomic references (CoL, EoL, GBIF).

**Additional file 7**. List of canonical scientific names which include generic epithets correspond to multiple taxonomic entities, collected from four taxonomic references.

**Additional file 8**. List of species name entries with strain-like elements, collected from COCONUT and NPASS databases.

**Additional file 9**. List of species name entries with modified scientific names, collected from COCONUT and NPASS databases.

## Data Availability

All metadata of taxonomic databases and NP occurrence databases are accessible via following URLs: Catalogue of life: https://download.checklistbank.org/col/. Encyclopedia of life: https://opendata.eol.org/dataset/tram-807-808-809-810-dh-v1-1/. GBIF: https://hosted-datasets.gbif.org/datasets/backbone/current/. NCBI taxonomy: https://ftp.ncbi.nih.gov/pub/taxonomy/. COCONUT: https://coconut.naturalproducts.net/download. IMPPAT: https://cb.imsc.res.in/imppat/. LOTUS initiative: https://zenodo.org/record/7534071. NPASS: https://bidd.group/NPASS/downloadnpass.html. PhyloSophos is publicly available at GitHub https://github.com/mhcho4096/phylosophos.
